# Online clinical reasoning assessment with the Script Concordance test: a feasibility study

**DOI:** 10.1186/1472-6947-5-18

**Published:** 2005-06-20

**Authors:** Louis Sibert, Stefan J Darmoni, Badisse Dahamna, Jacques Weber, Bernard Charlin

**Affiliations:** 1 Department of Urology and Department of Medical Education, Rouen University Hospital, 1, rue de Germont 76031 Rouen Cedex, France; 2 CISMeF, Rouen University Hospital, France & GCSIS, Perception System Information Lab FRE CNRS 2645, France, CISMEF & GCSIS, 1, rue de Germont, 76031 Rouen Cedex, France; 3 Unit of Research and Development in Medical Education, Faculté de Médecine-Direction, University of Montreal, CP 6128, succursale centre-ville, Montreal, Quebec H3C 3J7, Canada

## Abstract

**Background:**

The script concordance (SC) test is an assessment tool that measures capacity to solve ill-defined problems, that is, reasoning in context of uncertainty. This tool has been used up to now mainly in medicine. The purpose of this pilot study is to assess the feasibility of the test delivered on the Web to French urologists.

**Methods:**

The principle of SC test construction and the development of the Web site are described. A secure Web site was created with two sequential modules: (a) The first one for the reference panel (n = 26) with two sub-tasks: to validate the content of the test and to elaborate the scoring system; (b) The second for candidates with different levels of experience in Urology: Board certified urologists, residents, medical students (5 or 6^th ^year). Minimum expected number of participants is 150 for urologists, 100 for residents and 50 for medical students. Each candidate is provided with an individual access code to this Web site. He/she may complete the Script Concordance test several times during his/her curriculum.

**Results:**

The Web site has been operational since April 2004. The reference panel validated the test in June of the same year during the annual seminar of the French Society of Urology. The Web site is available for the candidates since September 2004. In six months, 80% of the target figure for the urologists, 68% of the target figure for the residents and 20% of the target figure for the student passed the test online. During these six months, no technical problem was encountered.

**Conclusion:**

The feasibility of the web-based SC test is successful as two-thirds of the expected number of participants was included within six months. Psychometric properties (validity, reliability) of the test will be evaluated on a large scale (N = 300). If positive, educational impact of this assessment tool will be useful to help urologists during their curriculum for the acquisition of clinical reasoning skills, which is crucial for professional competence.

## Background

The primary goal of medical teaching programs is the acquisition of clinical competence. Although a sound knowledge base, clinical and interpersonal skills are vital for a doctor; clinical reasoning represents a major component of clinical competence. Reasoning in the medical profession is much more than simple applications of knowledge, rules and principles. A significant part of a doctor's competence relies on the capacity to deal with uncertainty. In a clinical encounter, not all the data required to solve a problem are available. These data must be retrieved in order to formulate the problem and then solve it. Furthermore, problems can be confusing, contradictory and ill-defined [1], and are often characterized by imperfect, inconsistent or even inaccurate information. The capacity to reason in the context of uncertainty and to solve ill-defined problems is a hallmark of professional competence in medicine.

Traditional tools for assessing clinical reasoning, i.e. multiple choice questions (MCQ) correctly and reliably test the ability of students to apply well-known solutions to well defined problems. Test formats based on written simulations of clinical problem solving have repeatedly shown the puzzling fact that experienced clinicians judged competent by peers, often perform slightly better, and sometimes worse than clinicians with intermediate levels of experience (end-of training residents, for instance) [2]. Other important limitations of this type of assessment are difficulties of standardization, objectivity of scoring, and practicability for large groups of examinees. A further difficulty with assessment on ill-defined problems is that, as shown in medicine, in similar situations professionals do not collect the exact same data and do not follow the same reasoning patterns [3]. They also show substantial variation in performance as regards any particular real or simulated case [4]. Furthermore, most current performance-based methods of professional competence assessment (e.g. Objective Structured Clinical Exams) [5] are measures of behaviour.

At a time when cognitive psychology has become the major conceptual framework in educating for professions [6], it is necessary to add to these methods a way to assess reasoning cognition. It is also necessary to measure the process instead of its outcome. The adaptation of cognitive psychology script theory [7,8] to the characteristics of reasoning in the health professions provides a promising way to build a theory-based assessment tool. This theory implies that in order to give meaning and to act adequately to a given situation, professionals activate goal-directed knowledge structures relevant to the situation. These structures, named scripts, are used to actively process information to confirm or eliminate hypotheses, or management options [8]. According to this theory, reasoning is performed with a series of qualitative judgments. Each of these judgments can be measured and compared to those of a reference panel of experienced practitioners. This provides a method of assessment of reasoning on ill-defined problems and in contexts of uncertainty [9]. This method is called the script concordance approach.

The approach is based on three principles, each concern one of the following three components [10] inherent to any test: 1) the task required of examinees represents an authentic clinical situation and is described in a vignette. This vignette does not contain all the data required to provide a solution and several options (diagnosis, management or attitude) should be considered. 2) The response format is in accordance with what is known based on the clinical reasoning process [3,4]. A Likert scale, measuring the judgments that are constantly made within this process, retrieves examinees' answers. 3) The scoring method takes into account variation of answers among jury members. Credits on each item are derived from the answers supplied by a panel of reference. The method to build the tool is described in detail later in the article.

This tool has been used to date primarily in medicine. Previous studies have documented the reliability, the validity of the test and the validity of the scoring process [11-15]. Finally, research findings have shown that SC test has another advantage for a testing method of being relatively easy to construct and easy to administer. Relatively modest resources are required to develop the procedure. Furthermore, the test is standardized, scoring is straightforward, and the test is machine scorable and can be computer-based. Nevertheless, experience of SC test on a large population remains limited. No research, to our knowledge, has yet been conducted throughout an entire country.

Information and communication technologies (ICT) are gradually becoming a central part of medical education; in particular, the French Medical Virtual University consortium [16] of 20 French medical schools out of 31 (URL: ) has the objective of sharing experiences throughout the country using ICT to support new pedagogical approaches for medical students, including standardized assessment tools such as Objective Structured Clinical Examination (OSCE) and SC test. The elaboration of an Internet tool allowing administration of SC test on a very large number of candidates, with automatic correction and feedback, should optimize the potential of this new assessment approach. Further extensive research is required to verify psychometric properties and to assess the educational impact of the test when administered on the Internet. Its diffusion on a large scale should permit to confirm its utility as a strategy for investigating the process of decision making within the health professions.

Over the past several years, attention has been extensively focused on the development of computer-based testing in medical school curricula [17,18] and for national licensure examination [19,20]. Reported Web-based assessments have included short-answer, multiple-choice questions, extended matching questions [21], case simulations [120] and more recently, standardised patients [22]. To our knowledge, computer-based assessment with the SC test has never previously been described.

In this context, the purpose of this paper is to describe the development of a Web site to promote an on line assessment course of clinical decision making in contexts of uncertainty with the SC test. This feasibility study was conducted in the field of urology.

## Methods

The design of this web-based educational project has relied on a multidisciplinary approach. Content of the test was developed hand in hand with Web site designers, with close cooperation between each specialist of the team (medical educators, medical informatics specialist, and research engineer). We adhere to the main principles of practical guidelines for developing effective educational website [23]

### Development of the Web site

The TCS online Web site respects the traditional three-tier architecture used in Web database applications. The development process is based on standards models of Information Systems Engineering, i.e. a life cycle following 4 main phases: 1) the feasibility study; 2) the need analysis and specifications; 3) the detailed conception; and 4) the implementation (code writing, tests and validation). Medical educators were involved in each step of the development process.

**Figure 1:**
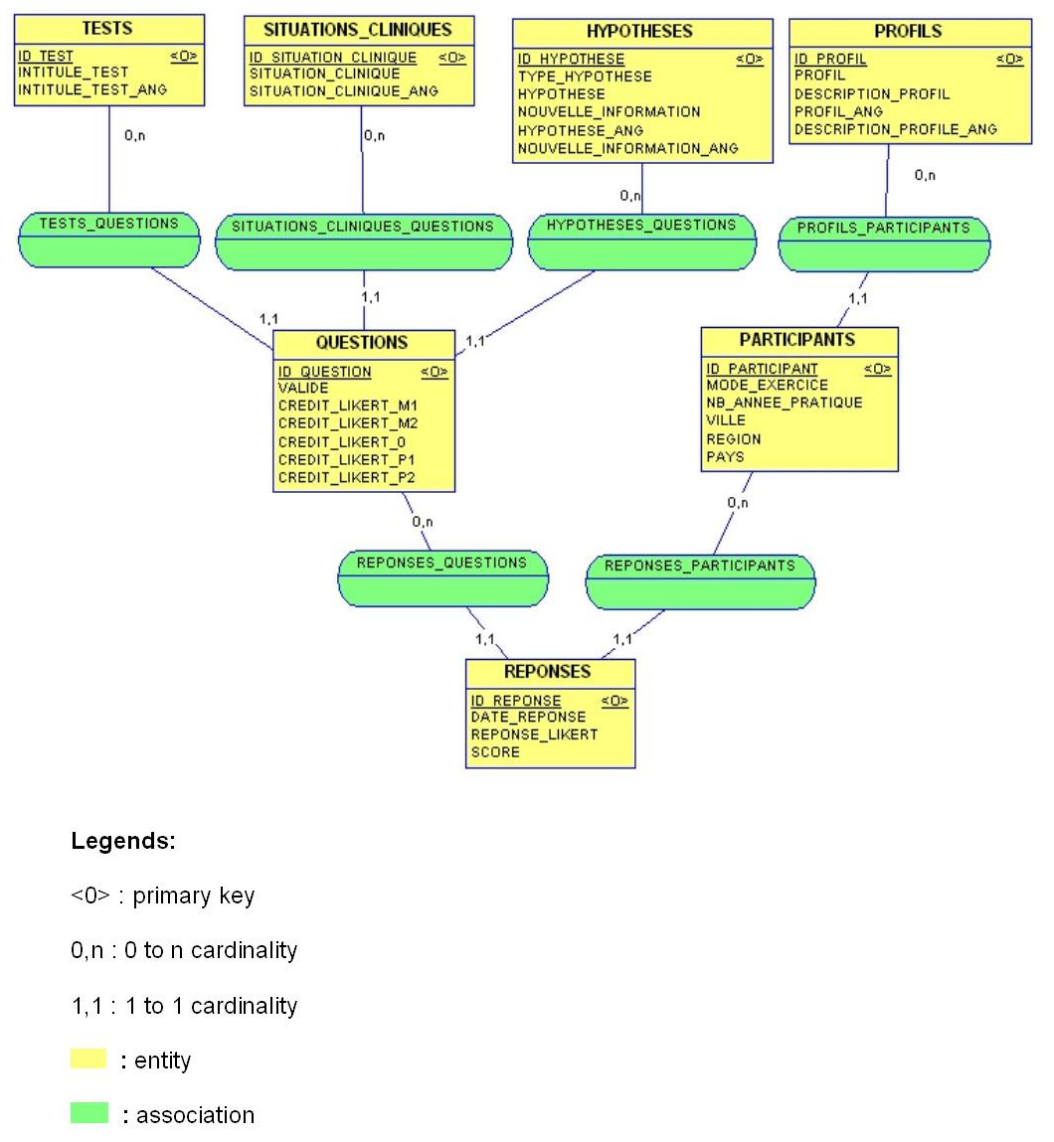
Conceptual Data Model for the SC test online.

The feasibility study assessed the suitability of the SC test for a Web-based approach. We then verified that technical and managerial conditions were met to implement the SC test. Technical choices, such as the use of MySQL 4.0.18 as a Database Management System and PhP 4.3.4 as programming language, were decided in this step. As regards the needs and rules analysis of the SC test, we have produced functional specifications and technical specifications. We have evaluated concordance scripts procedures, and established the behaviour of the SC test in a manner that is achievable through the web technology. The description of the resulting relational database has been specifically designed for the level of detailed conception, database diagrams, Entity-Relationship Modelling, and Physical Model. We have also addressed, but in a less formal way, Structure Chart Diagrams with the aim of designing the processes structure, and Layout Diagrams for the Web interface. Finally, during the implementation process, the focus was placed on prototypes, which were sufficiently well engineered for use in the Internet environment. A total of 115 SC test items were then included in the database. After the coding phase and integration tests, the validation step assessed that the SC test was in fact operational (see Figure 1).

### Development of the SC test

A bank of SC test items for urology has been developed since May 2001 by researchers from the Rouen University Hospital and the Faculty of Medicine of the University of Montreal (LS and BC) according to the methodology previously described by Charlin et al. [9]. Two faculty members were asked to a) describe clinical situations representative of urology practice and based on major educational objectives of urology training programmes; b) specify for each situation, the questions they would ask and the actions they would take to arrive at a diagnosis or decide on the adequate management of the patient. Test items were built using the material obtained at this stage.

The clinical situations are presented in short vignettes. The description of the situation must be complex enough to be challenging for the level of training that has to be assessed (urology residency, in this context). They must not contain all the data to provide a unique solution. Each vignette is followed by a series of related items. The item format differs with the objective of assessment (diagnosis, investigation, or treatment). Each item consists of three parts. The first part includes a diagnostic hypothesis, an investigative action or a treatment option. The second presents new information (e.g. a clinical data, imaging study or laboratory test result) that might have an effect on the diagnostic hypothesis, investigative action or treatment option. The third part is a 5-point Likert-type scale (see illustration of the 3 formats in Table 1). Each item was built so that a reflection was necessary to answer it. It was also clearly specified in the instructions for each participant that within the vignettes, each item is independent of the others. Hypotheses or options change for each question. The instrument used for our Web site (115 items) was created using items from the bank. An example of items from the diagnostic section of the test is illustrated in Table 2.

**Table 1:** Illustration of questions and answering grid format. The item format varies with the object of assessment (e.g. diagnostic, investigation, treatment).

**For diagnostic knowledge assessment**		
If you were thinking of	And then you find	This hypothesis becomes

*(A diagnosis hypothesis)*	*(A new clinical information, an imaging study or a laboratory test result)*	-2 -1 0 +1 +2

-2 the hypothesis is almost eliminated -1 the hypothesis becomes less probable 0 the information has no effect on the hypothesis +1 the hypothesis is becoming more probable +2 it can only be this hypothesis		

**For investigation knowledge assessment**		

If you were considering to ask	And then you find	This investigation becomes

*(A diagnostic test)*	*(A new clinical information, an imaging study or a laboratory test result)*	-2 -1 0 +1 +2

-2 contra-indicated totally or almost totally -1 not useful or even detrimental 0 nor less nor more useful +1 useful +2 absolutely necessary		

**For treatment knowledge assessment**		

If you were considering to prescribe	And then you find	The relevance of this treatment becomes

*(A therapeutic option)*	*(A new clinical information, an imaging study or a laboratory test result)*	-2 -1 0 +1 +2

-2 contra-indicated totally or almost totally -1 not useful or even detrimental 0 nor less nor more useful +1 useful +2 necessary or absolutely necessary		

**Table 2:** Example of a clinical vignette with items from the diagnostic section of the SC test. Clinical Vignette: A 25 year-old male patient is admitted to the emergency room after a fall from a motorcycle with a direct impact to the pubis. Vital signs are normal. The X-ray reveals a fracture of the pelvis with a disjunction of the pubic symphysis.

If you were thinking of	And then you find	This hypothesis becomes				
Urethral rupture	Urethral bleeding	-2	-1	0	+1	+2
Retroperitoneal bladder rupture	Bladder distension	-2	-1	0	+1	+2
Urethral rupture	Upward and bulging prostatic apex at the digital rectal examination	-2	-1	0	+1	+2
Intra-peritoneal bladder rupture	Spontaneous micturition after the accident	-2	-1	0	+1	+2
Urethral rupture	Perineal haematoma	-2	-1	0	+1	+2

### Scoring process

The aggregate scoring method [24] used with the test reflects the variability experts demonstrate in their reasoning processes. Credits on each item are derived from the answers given by a panel of reference. The credit for each answer is the number of panel members that have provided that answer, divided by the modal value for the item. For example, if on an item, six panel members (out of 10) have chosen response +1, this choice receives 1 point (6/6). If three experts chose response +2, this choice receives 0.5 (3/6), and if one expert chose response 0, this choice receives 0.16 point (1/6). The total score for the test is the sum credits obtained on all items. This score is then divided by the number of items and multiplied by 100 to get a percentage score.

### Participants

#### Reference panel

A total of 26 urologists from University, general and private practice volunteered to participate to this study. They comprised of a relatively broad sample of urologists with variability in demographics, training background and level of experience, thus constituting an appropriate population for development of a norm referenced database of performance and for study of the assessment method. After their completion of the test, members of reference panel were asked to identify the items they found confusing or not relevant. Eighteen items were then excluded. Final SC test submitted online to candidates was made up of 97 items and 17 clinical situations.

#### Candidates

In order to assess psychometric properties of the SC test, participants are identified according to their level of experience in Urology. Three groups of participants with different levels of experience in Urology are recruited on a voluntary basis: Board certified Urologists, Urology residents, and medical students (5 or 6^th ^years). Inclusion criteria to participate in the SC test were: for the Board certified Urologists, to be member of the French Urology Association (AFU) with an access to the SC Test on line via the Web site of AFU (URL: ); for the Urology residents, to be trainees of the National Urology Training Program; and for the medical students, to have a rotation in Urology during the past six months before the SC test. Minimum expected number of participants is 150 for the Board certified Urologists, 100 for the residents and 50 for medical students. These figures will provide a robust statistical analysis.

## Results

The "SC test online" is located on the Web site of Rouen University Hospital (URL: ), which was created in 1995. A secure Web site was created in two months (February & March 2004) for the two populations (reference panel and candidates). The evaluation system can be accessed via any computer system with a standard Web browser. A userid/password is required for each individual to enter the "SC test online" Web site. A second userid/password is necessary for the reference panel. The Web site has been operational since April 2004.

French Urologists and residents have never completed an assessment with an online system prior to this experience. An information seminar for the reference panel regarding the SC test online was presented in June during the Annual Seminar of the French Society of Urology. All members of the reference panel completed the SC test during the following two months. The Web site has therefore been available for the participants since September 2004. At the end of February 2005, 195 candidates had already passed the SC test online. Among them, 92 participants did not answer all the items and only partially completed the SC test.

**Figure 2:**
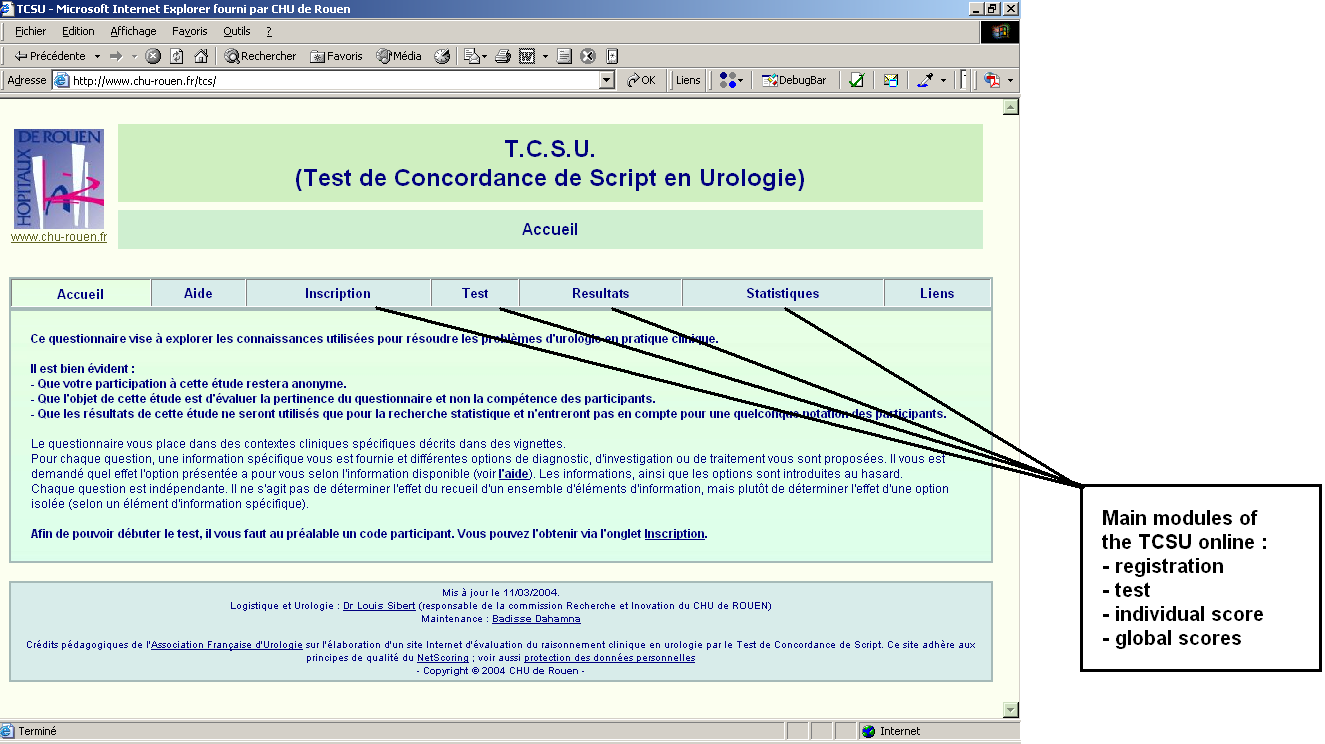
Illustration of the SC test on line home page (URL: )

**Figure 3:**
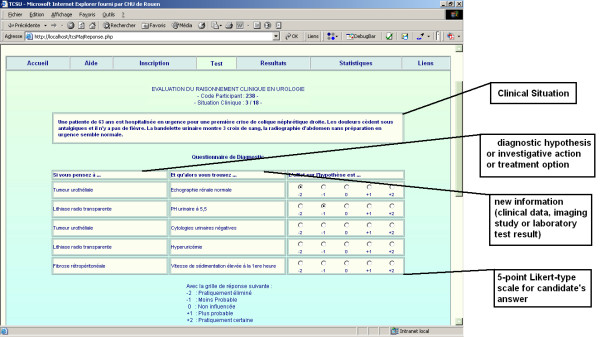
Illustration of the SC test on line module page.

**Figure 4:**
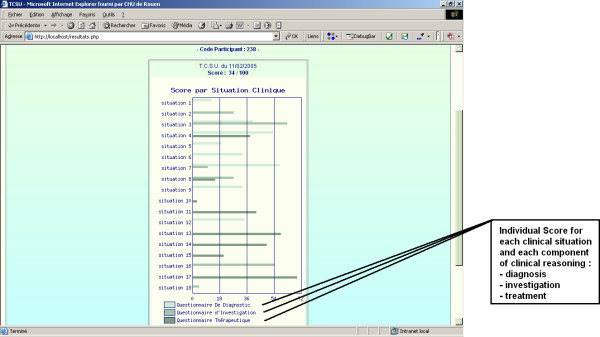
Individual score module page of the SC test on line.

The SC test online home page (see Figure 2) contains several modules: one to register as a candidate or as a member of the reference panel, another to pass the test, another to obtain the individual test score for each candidate and another to obtain the global scores by groups or by demographic data. The home page contains a summary of the SC test principles as well as the instructions for the participants. The test module (see Figure 3) consisted of 97 items and 17 clinical situations. The estimated time to perform the test was 65 minutes. The individual score module (see Figure 4) allow for each candidate to access their global score but also his/her score for each clinical situation and each component of clinical reasoning (diagnosis, investigation and treatment).

Participants' recruitment was rapid; In six months, 80% of the target figure for the Urologists, 68% of the target figure for the residents and 20% of the target figure for the student passed the test online. During these six months, no technical problem was encountered in this web site, which is 24/7 since February 1995. Two e-mails were sent due to difficulties in subscription (in 195 participants).

## Discussion

Efficient and meaningful evaluation of clinical competence is critical to the professional development of trainees in medical training programmes. Current paper-based evaluation instruments have numerous limitations: difficulty in data analysis, significant delays in identifying problem trends and poor user compliance. ICT now offer the possibility to validate learning and assessment tools on a large scale over a relatively short period of time. Creation of the Web site was achieved in a very short period of time (two months). Twenty-six members of the reference panel completed the test online in two months. Two-thirds of the expected number of participants has already been included in the subsequent months: i.e. 80% of the Board certified Urologists and 68% of the expected number of the residents passed the SC test online during a six months period.

A useful assessment tool should have a series of qualities in terms of feasibility and acceptability. In our study, validation tasks and tasks related to elaboration of answer keys of the SC test seem to be well accepted by experts, as demonstrated by the rapidity of inclusion of the expected number of reference panel's members, without particular difficulty. In addition, in a SC test, the sometimes-lengthy discussions required to arrive at consensus answers in other testing formats is not required. Furthermore, practicing urologists and residents appear to enjoy completing a test that is close to real clinical reasoning, as demonstrated by the numbers of Urologists and residents who passed the SC test via our Web site despite their lack of experience of online evaluation system.

The technical concept of the SC test online enabled an automatic auto evaluation of the results immediately after the online examination. In addition to being able to inform participants of their results in real time, it will be also possible to reduce correction time and personnel resources. The facility of the Internet to implement SC test online is obvious. The administration of assessment tools over the Internet offers many opportunities to address suboptimal prescription of physicians in practice. One can imagine test modules available on electronic campuses. The physician would be able to select any module, pass the SC test, and either succeeds (sufficiently high score when compared with the reference panel) and obtains training credits or fails. In this case he may receive a notification of his/her zones of weakness with hyperlinks to relevant references. He can then later undertake training specifically focused on areas of weakness.

The use of the Internet tool should permit to quickly evaluate psychometric properties of the test on a large scale (N = 300). The statistical analysis (construct validity, reliability) of SC test in Urology will be submitted to BMC Medical Education in the near future. Therefore, with the use of the Internet, this should facilitate a more accurate approach regarding the utility and the educational impact of this tool in the overall urology training programmes. If positive, SC test will be useful to assist urologists during their curriculum for the acquisition of clinical reasoning skills, which is an major component of professional competence

The Script Concordance approach is designed to measure the quality of a set of cognitive operations or knowledge structures by comparing a participant's problem representation, judgements and choices to those of an experienced clinicians group. The test can be used in situations where there is no consensus among experts, in the literature or in practice. Finally, SC test offers the opportunity of a wide range of assessment of decision-making skills in contexts where evidence-based medicine cannot be applied. Nevertheless, any given examination instrument has its limitations. Professional competence in medicine is a multi-dimensional entity and cannot be adequately measured by a single assessment method. SC test should be used in complement with what could be considered as the optimal tool of evidence-based factual knowledge assessment, i.e. rich-context MCQ as used by the National Board of Medical Examination in the United States [25]. These concepts underline the current need to promote this type of Web site.

Nevertheless, this Web site does present some technical limitations, i.e. multimedia resources (sounds, images, and videos), which were not previously developed. In fact, the primary goal of our study is to include a maximum number of participants with the Internet tool. These new functionalities will be implemented in the first semester of 2005 and will enhance the differences between the SC test on paper vs. the SC test on the Internet.

Moreover, despite the fact that two-thirds of expected number of participants has already been included in our study, only 20% of the target figures for the students passed the SC test online. Feedback possibilities of our Web site will permit us to focus on the recruitment on this specific population. One other relevant point of discussion is the fact that 41% of the participants did not entirely complete the SC test. Improvement in the completion rate of SC test online is required. To address this issue, we organized training sessions using video conferencing to teach participants how to log on to the system and complete the SC test. The high number of test questions (97) could also possibly explain the rate of completion. Optimisation of the SC test content, with a smaller number of items is necessary. Medical education literature suggests that an assessment tool is often considered to be sufficiently reliable when its reliability coefficient reaches a value of 0.80 [2]. Previous studies on SC test have repeatedly demonstrated that a reliability coefficient value of 0.80 could be reached with 60 to 70 items [9,11-15]. In the near future, an early stage in evaluating this Web site will be to verify that the exam format will measure the examinee's clinical reasoning and not their comfort level or confidence with the technology.

Several other Web sites of SC tests are also currently being developed in Bordeaux, France and in Montreal, Canada (URL: ). The Bordeaux Web site is focusing on CME (URL: ). To our knowledge, no report of SC test online experience has ever been previously published. Montreal, Bordeaux and Rouen, are presently constructing a consortium to promote online clinical reasoning assessment using SC tests. Furthermore, the number of participants recruited over a short period of time in this feasibility study has encouraged us to extend this experiment to other medical disciplines. The Rouen Medical School is one of the founders of the FMVU consortium. One of the FMVU aims is to adapt "SC test online" for formative evaluations of clinical reasoning to prepare medical students to the new pre-residency examination in France. An online SC test prototype was recently designed and is now freely accessible (URL: ).

## Conclusion

The feasibility of the web-based SC test was successful as two-thirds of the expected number of participants was included within a six months period. The creation of a Web-based instrument for evaluation of decision-making skills and clinical reasoning should be a potentially powerful instrument for evaluating French Urology training programmes and making changes to improve the educational experience in a timely and efficient manner. Virtual medical education initiative such as this Web site warrant consideration in the current context of medical training programmes harmonization at international level.

## Competing interests

The author(s) declare that they have no competing interests.

## Authors' contributions

LS was the main investigator of the project and drafted the manuscript. SJD and BD developed the Web site, were responsible for data acquisition and revised the manuscript. Critical revisions were carried out by JW. BC was the inventor of the SC test, had the original idea of on line assessment of clinical reasoning with the SC test. He was involved in conceiving and planning the study. Funding was obtained by LS.

## Pre-publication history

The pre-publication history for this paper can be accessed here:


